# Natural Intestinal Protozoa in Rodents (Rodentia: Gerbillinae, Murinae, Cricetinae) in Northwestern Iran

**Published:** 2017

**Authors:** Mehdi MOHEBALI, Zabiholah ZAREI, Khadijeh Khanaliha, Eshrat Beigom KIA, Afsaneh MOTAVALLI-HAGHI, Jaber DAVOODI, Tahereh REZAEIAN, Fathemeh TARIGHI, Mostafa REZAEIAN

**Affiliations:** 1. Dept. of Medical Parasitology and Mycology, School of Public Health, Tehran University of Medical Sciences, Tehran, Iran; 2. Center for Research of Endemic Parasites of Iran (CREPI), Tehran University of Medical Sciences, Tehran, Iran; 3. Research Center of Pediatric Infectious Diseases, Rasool-e-Akram Hospital, Iran University of Medical Sciences, Tehran, Iran; 4. Dept. of Veterinary Parasitology, Islamic Azad University, Zanjan Branch, Zanjan, Iran

**Keywords:** Rodent, Intestinal protozoa, Iran

## Abstract

**Background:**

Majority of parasitic infections in rodents have zoonotic importance. This study aimed to determine the frequency and intensity of intestinal protozoa infections of rodents including *Meriones persicus, Mus musculus* and, C*ricetulus migratorius*.

**Methods:**

This survey was conducted in Meshkin Shahr district in northwestern Iran from Mar. to Dec. of 2014. Intestinal samples of 204 rodents including *M. persicus* (n=117), *M. musculus* (n=63) and C. *migratorius* (n=24) were parasitologically examined. Formalin-ether concentration method was done for all of rodents stool samples and observed with light microscope. All of suspected cases were stained with trichorome staining Method. Cultivation in dichromate potassium 2.5% was carried out for all of coccidian positive samples. Acid fast and aniline blue staining methods were used for detecting of coccidian oocysts and intestinal microsporidial spores, respectively.

**Results:**

About 121(59.3%) of the caught rodents were generally infected with intestinal protozoa. *Entamoeba muris* 14(6.9%), *Trichomonas muris* 55(27.0%), *Chilomastix betencourtti* 17 (8.3%), *Giardia muris* 19(9.3%), *Eimeria* spp. 46(22.5%)*, Isospora* spp. 4(2%) and *Cryptosporidium* spp. 1(0.5%) were found from the collected rodents. Microsporidian spores were identified in 63 (31%) out of the 204 collected rodents using aniline blue staining method.

**Conclusion:**

Since some of the infections are zoonotic importance thus, control of rodents can be decreased new cases of the parasitic zoonoses in humans.

## Introduction

Parasitic infection including protozoa and helminthes in rodents are of special interest because of the role of rodents as reservoirs of many important parasites of man (1). All rodents are susceptible to protozoan infection. Some of these protozoa may be zoonotic. Although *Giardia muris* has a limited host and its transmission to human from laboratory rodents has not been reported, care should be taken with *Giardia* and *Cryptosporidium* spp. (2, 3).

A form of *Giardia muris* has been observed in the golden hamster, mice, and rats. Infection is usually subclinical but the animal’s exhibit weight loss, hair bristling, bloating with meteorism (4).

*Trichomonas muris* is common parasite that detected in rodents, as mice. Its pathogenesis in mice is unclear. Although *T. muris* suspected to be non-pathogenic (5), diarrhea and anorexia have been reported as sign of *T. muris* infection (6).

Some possible protozoa infection in rat and mice are *Chilomastix Bettencourt, Entamoeba muris, Cryptosporidium muris, Giardia muris, Cryptosporidium parvum, Trichomonas muris, Hexamita muris, Eimeria* spp., *Spironucleus muris* that some of them are described as mildly pathogen (2, 3, 7). *Eimeria* spp. has been reported as common protozoa parasites in rabbits and *Balantidium coli* identified in guinea pigs (8).

Although helminthic fauna and *Leishmania* infection in rodents as a host of visceral Leishmaniosis in Meshkin Shahr district were reported (9,10, 11), there is no data on protozoa infection in this area.

This study aimed to determine frequency and intensity of intestinal protozoa infections of rodents including *Meriones persicus, Musmus culus* and, C*ricetulus migratorius* (gray hamster) from Meshkin Shahr district, northwestern Iran.

## Materials and Methods

**Fig. 1:**
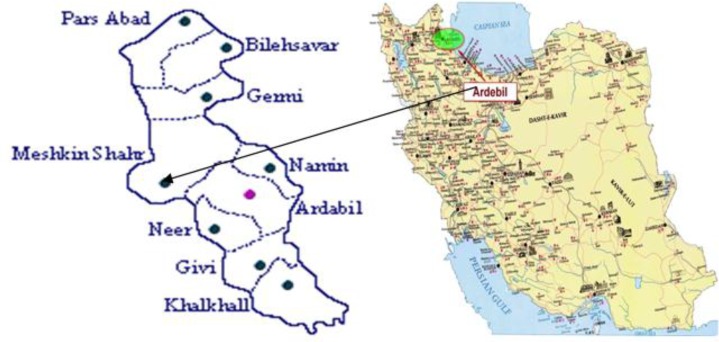
Geographical situation of Meshkin Shahr district

Meshkin Shahr is located in the northwest of Iran in Azerbaijan, It covers an area of approximately 1530 km^2^ and its population is estimated to be 237585, among whom 29.7% are settled in urban areas and 70.3% live in 323 rural areas. It is the nearest city to the Sabalan high mountain. The weather of this city and the district of Meshkin Shahr is moderate mountainous (12) (Fig.1).

### Sample collection

From Mar. to Dec. of 2014, intestinal samples from 204 trapped rodents including *M. persicus* (no.117), *M. musculus* (no.63) and *C. migratorius* (no.24) were collected by Sherman method, live animal Traps from northwest of Iran (13). This study was approved by the Research Ethical Review Committee of Tehran University of Medical Sciences, Tehran, Iran with No: 22943.

All of rodents stool samples were preserved in formalin 10%, PVA solution and saline solution in Meshkin Shahr research station and transferred to the Department of Medical Protozoology and Mycology, School of Public Health, Tehran University of Medical Sciences. Formalin-ether concentration method carried out for all of the samples and samples observed with light microscope with 400× magnification. Cultivation in dichromate potassium 2.5% was carried out for all of coccidian positive samples that collected in saline solution.

All of suspected cases were stained with trichrome staining method (14). The slide was mounted using Canada balsam and observed under 1000× magnification. Detection of intestinal protozoa was based on morphological characteristic of specific protozoa. Analysis was performed using Excel 2007. Formalin-ether concentration method carried out and smears were prepared from pellet of all samples. The slides were dried at room temperature for 5 min after methanol fixation all of samples were stained with modified acid-fast staining method (15), finally all of slides were observed with under light microscope 1000× magnification. Samples smear were prepared and after drying and methanol fixation, aniline blue staining method carried out according to Ryan method (16). All of samples were observed with 1000× objective and evaluate for detecting microspore spores.

## Results

From 204 the caught rodents, 127(62.1%) were male. In general, 121 (59.3%) of rodents were infected with protozoa including *M. persicus* 88 (75.2%), *M. musculus* 20 (31.2%) and 13 (54.2%) grey hamster.

Infection rates of protozoa in male *M. persicus*, *M. musculus* and grey hamster were 52.3%, 60%, and 84.6%, respectively that they were more than female as 47.7%, 40% and 15.4%, respectively. Generally, the prevalence of protozoa infection in male rodents 69(57%) was more than females 52(43%).

Prevalence of Intestinal protozoa was as below: *E. muris* 14(6.9%), *T.muris* 55(27.0%), *Chilomastix betencourtti* 17(8.3%), *G. muris* 19(9.3%) and from coccidian group: *Eimeria* spp. 46(22.5%)*, Isospora* spp. 4(2%) and *cryptosporidium* spp. 1(0.5%) (Table 1).

**Table 1:** Frequency of Intestinal protozoa among 204 trapped rodents from Meshkin Shahr, Ardabil Province in 2014

**Parasite**	***Meriones* Infected no**	***persicus* %**	***Mus* Infected no**	***musculus* %**	***Cricetulus-migratorius* Infected no**	**%**	**Total Infected no**	**%**
*Entamoeba muris*	12	5.9	1	0.5	1	0.5	14	6.9
*Trichomonas muris*	40	19.7	7	3.45	8	3.94	55	27.0
*Chilomasix Bettencourt*	16	7.9	0	0	1	0.5	17	8.3
*Giardia muris*	15	7.4	1	0.5	3	1.5	19	9.3
*Eimeria* spp	37	18.23	7	3.45	2	0.98	46	22.5
*Cryptosporidium* spp	1	0.5	0	0	0	0	1	0.5
*Isospora spp*	4	2	0	0	0	0	4	2
Microsporidia Spores	48	23.64	11	5.42	4	1.97	63	31.03

Prevalence of protozoa infection in *M. persicus* 88 (72.8%) was more than *M. musculus* 20 (16.5%) and *C. migratorius* 13(10.7%).

**Fig. 2:**
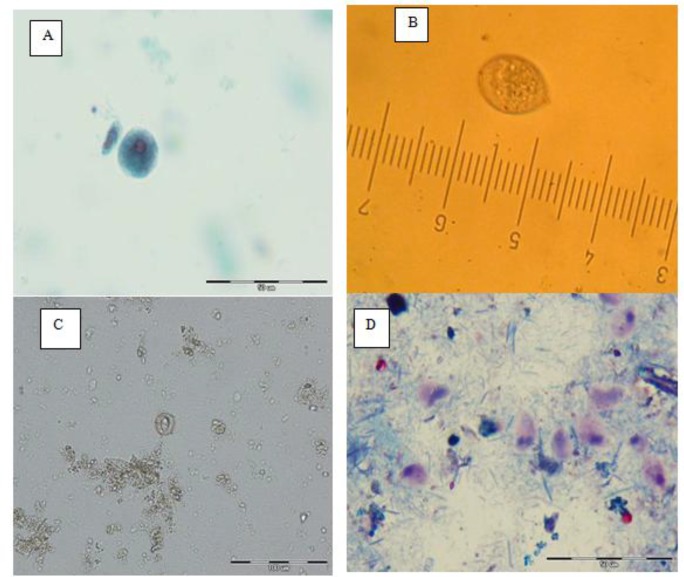
A: Trophozoite of *E. muris* and *T. muris* in trichorom staining method (1000×); B: An unsporulated oocyst of coccidia in a wet mount sample (400×); C: appearance of *Eimeria* spp after cultivation in dichromate potassium (400×); D: *Cryptosporidium* spp in acid-fast staining method (1000×), in trapped rodents from Meshkin Shahr in 2014.

Microspora spores were identified in 63(31.03%) of all samples were stained by the aniline blue staining method. In aniline blue staining method was used for detecting *Microspora*, ovoid, transluminant spores were observed with 0.7–1.2 μm size. The spores had a belt-like strip in the middle or at the end of body. Trophozoite of *E. muris* and *T. muris* stained with trichrome staining method with1000× magnification are shown in (Fig. 2, A) and an unsporulated oocyst of coccidia in a wet mount sample with 400× magnification are shown in (Fig. 2, B)

The result of coccidia cultivation in dichromate potassium 2.5% is shown in Fig. 2, C. In each oocyst four sporocysts and in each sporocyst two sporozoites exist, and finally *Eimeria* spp. diagnosis was made.

The result of acid-fast staining method showed partial acid-fast positive cases with the size of almost 4 μm, in a sample that belongs to a male *M. persicus* and finally, *Cryptosporidium* spp diagnosis was made (Fig. 2, D).

In general 121(59.3%) were positive that 63(52.1%) have just one parasite, 32(26.4%) two parasite, 15(12.4%) three, 8(6.6%) four, 2(1.6%) five, 1(0.8%) six, parasites.

The most common single protozoa were *Microspora* 24/63 and *T. muris* 19/63 and then *Eimeria* 13/63 and double infection were between *Eimeria* and *Microspora* (8/32), *T. muris* and *Microspora* spp. (8/32), *Trichomonas* and *Eimeria* (5/32).

## Discussion

This study was conducted to determine the prevalence of intestinal protozoa infection of rodents from Meshkin Shahr district, northwestern Iran.

In general, 121 (59.3%) of rodents were infected with protozoa. The most common protozoa were *T. muris* 27.0%, followed by *G. muris* (9.3%) and *E. muris* (6.9%). *T. muris* was the most common protozoa (8, 17, 18). Overall prevalence of intestinal parasites in rat in Arbil was 76% including *T. muris* with the higher incidence of 56%, *G. muris* 12%, *H. muris* 8% and the least infection percentage was 4% for *E. muris* (17). The result of our study is consistent with that.

Prevalence of intestinal protozoa was reported in the mice as follows: *S. muris* (46.2%); *G. muris* (46.2%); *T. muris* (53.8%); *T. minuta* (61.5%) and *E. muris* (84.6%), while in the rat colonies the prevalence of infection was higher: *S. muri*s (85.7%); *T. muris* (85.7%); *T. minuta* (85.7%) and *E. muris* (85.7%) (7). The high prevalence of parasitic infections was found in an animal house in Brazil. The prevalence rate of protozoa were: *T. muris* (80.0%), *G. muris* (66.0%), *E. muris* (20.0%), and *Eimeria* sp. (13.3%) (18).

Prevalence of protozoa infections have been reported in mice and rat, *Entamoeba* sp. (8.08%, 3.18%), *Giardia* sp. (0%, 0%), *Trichomona* sp. (8.88%, 1.58%), *Chilomastix* sp. (3.74%, 1.65%) *Spironucleus* sp. (0.08%, 0.19%) respectively in North America and Europe (19). Prevalence rate of protozoa infection in our study was higher than those have been reported from North America and Europe.

Overall, 37 mice (74%) from 50 Swiss-Webster mice were infected with at least one parasite. The highest prevalence was related to *S. muris* (64.8%) then follow by *G. muris* (27.01%), *T. muris* (21.6%) and the lowest prevalence rate was related to *Blastocystis* spp. (2.7%) (20).

*Encephalitozoon* sp. has been reported from rabbits, mice, guinea pigs and rats. The organisms are small, bipolar and rod-like. They occur singly or in clumps (4). Three strains (I, II, and III) are recognized in *E. cuniculi*, which, according to the host of the originally characterized isolates, are also designated “rabbit strain,” “mouse strain” and “dog strain” (21).

In the present study, microspora spores were identified in 63(31.03%) of all samples. *Encephalitozoon* sp. was found in (55%) of mice samples by parasitological method (22).

In our study from coccidia group: with the higher incidence of *Eimeria* spp 46(22.7%), followed by *Isospora* spp 4(2%) and 1(0.5%) *Cryptosporidium* spp were found. In concentrated pellets, some unsporulated oocysts were found and after cultivation in dichromate potassium and observation of pattern of four sporocysts and two sporozoites, *Eimeria* spp diagnosis was made and about *Isospora* unsporulated and sporulated oocysts were seen. *Eimeria* is the most parasites from coccidian group in rodents in our study and it was also described in some previous study (4, 23, 24).The overall prevalence of *Eimeria* spp in rabbits from pet shops and farms were 46.2% and 41.7%, respectively in Taiwan that is similar our result (23, 24).

*C. muris* has been reported from mice and the guinea pig. They are true coccidia whose developmental stages appear to take place on the surface of the host cell but not within the cell proper. In the case of *C. muris*, the parasite may be seen in large numbers in sections of the stomach and is a parasite of the peptic glands, but *C. parvum* has been reported in rat and found in the glandular structures of the small intestine of the mouse (4, 25). In this study, one *Cryptosporidium* spp positive was detected in a sample belongs to a *M. persicus*. Molecular investigation is needed for confirmation of *Cryptosporidium* species.

Protozoa infection in male rodents 69(57%) was more than female 52(43%). In the present study, there was no significant difference between male and female rodents in consistent with another others (26).

In a study, single parasitic infection was the highest (52%), followed by double infection (16%), and triple infection (8%) (17). In the present study, 63(52.1%) of rodents stool samples had just one parasite, 32(26.4%) two parasite, 15(12.4%) three, 8(6.6%) four, 2(1.6%) five, 1(0.8%) six parasites. Prevalence of single parasite was similar but double and triple infection was more than similar study in Arbil (17).

## Conclusion

Rodents as reservoirs of some important parasites in this area infected with some zoonotic parasites, hence control of these animals has an important role on prevention of public health problems.
